# Supplementary material for: The ADP-Heptose Biosynthesis Enzyme GmhB is a Conserved Gram-Negative Bacteremia Fitness Factor
Source: Infect Immun. 2022 Jun 28;90(7):e00224-22. doi: 10.1128/iai.00224-22 (PMC9302095; doi:10.1128/iai.00224-22)
Supplement: Supplemental file 2 — Fig. S1-S8; Tables S1-S3; description of Data Set S1. Download iai.00224-22-s0002.pdf, PDF file, 1.4 MB [file iai.00224-22-s0002.pdf]

SUPPLEMENTAL FIGURES

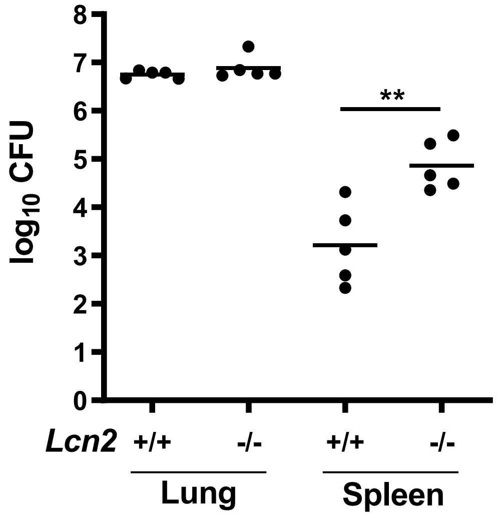

<u>**Supplemental Figure 1**: Lipocalin 2 restricts *K. pneumoniae* lung dissemination.</u> To model pneumonia, $1x10^6$ CFU of a library of *K. pneumoniae* transposon mutants was administered retropharyngeally to *Lcn2*$^{+/+}$ or *Lcn2*$^{-/-}$ mice. Mean log_{10} CFU is displayed for each organ at 24 hours post infection. **$p<0.001$ by unpaired t test. For each group, n=5 mice.

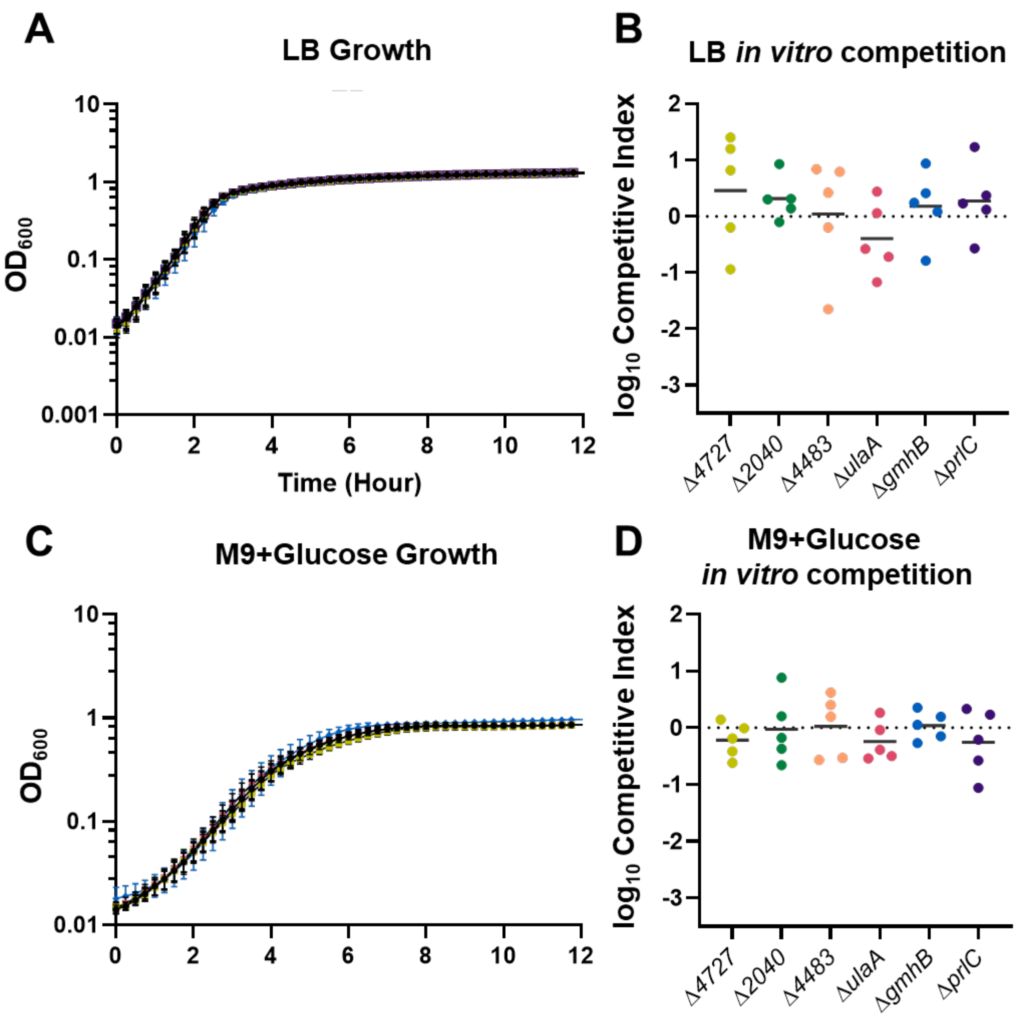

Supplemental Figure 2. GmhB and other factors of interest are not required for *K. pneumoniae* replication *in vitro*. KPPR1 or isogenic knockouts were inoculated to a starting concentration of 1×10^7 CFU/mL and monitored by optical density (OD_{600}) in LB (A) and M9 with 0.9% glucose (M9+Glucose; C). KPPR1 and each mutant were combined 1:1 at a concentration of 1×10^6 CFU/mL and incubated in LB (B) or M9+Glucose (D) and mean $\log_{10}$ competitive index compared to wild-type at 24 hours post inoculation is displayed (n=5). One-way ANOVA indicated no significant difference between strains for area under the curve after growth and one-sample t tests with a hypothetical value of zero showed no defect in competitive indices; for A and C, lines colors correspond to strains in B and D.

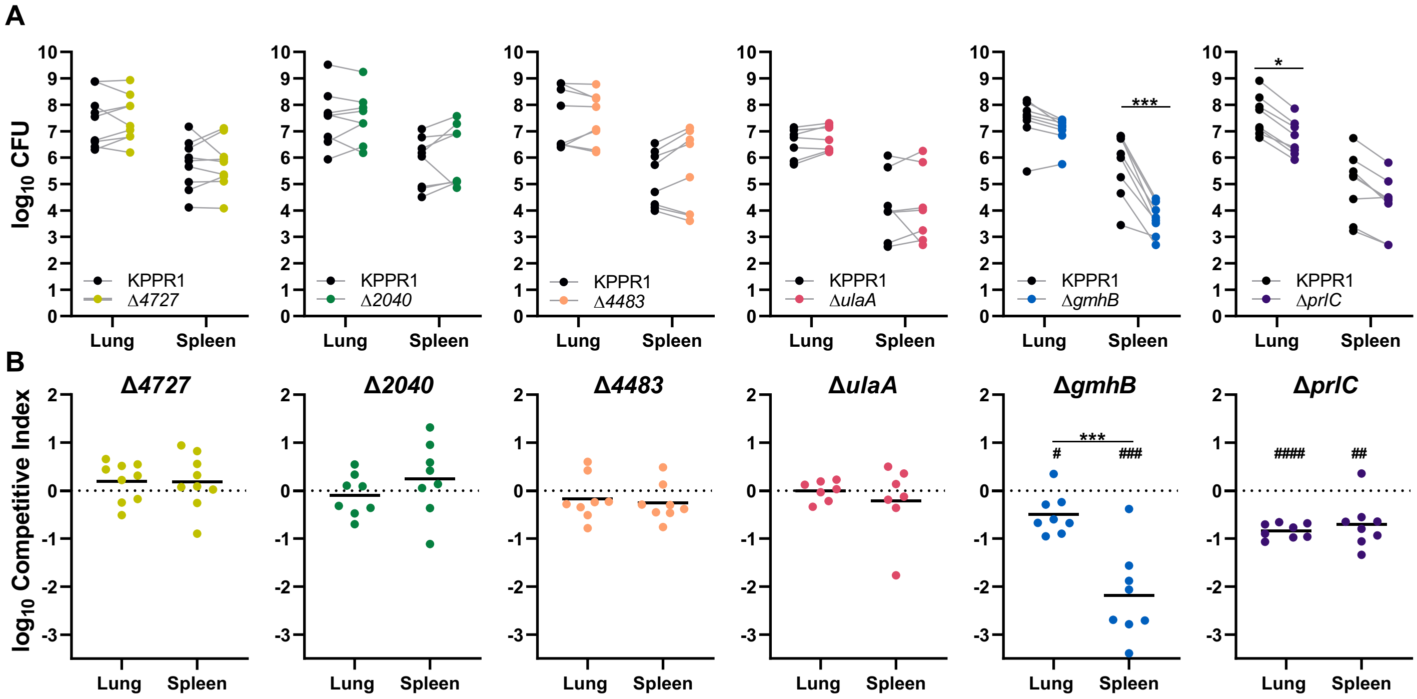

Supplemental Figure 3. Summary for *in vivo* validation of transposon insertion site sequencing (InSeq). Bacterial burden per organ from mice 24 hours after inoculation with 1:1 mixture of isogenic knockouts and KPPR1 ($1x10^6$ CFU total) administered in the pharynx of *Lcn2*$^{-/-}$ mice is shown (A), corresponding to the competitive indices in (B). *p<0.05, ***p<0.001 by unpaired t test; $^{\#}$p<0.05, $^{\#\#}$p<0.01, $^{\#\#\#}$p<0.001, $^{\#\#\#\#}$p<0.0001 by one sample t test with a hypothetical value of zero. For each group, n≥7 mice in at least two independent infections. All statistical tests were performed on log-transformed data. For each group, n≥7 mice across at least two independent infections.

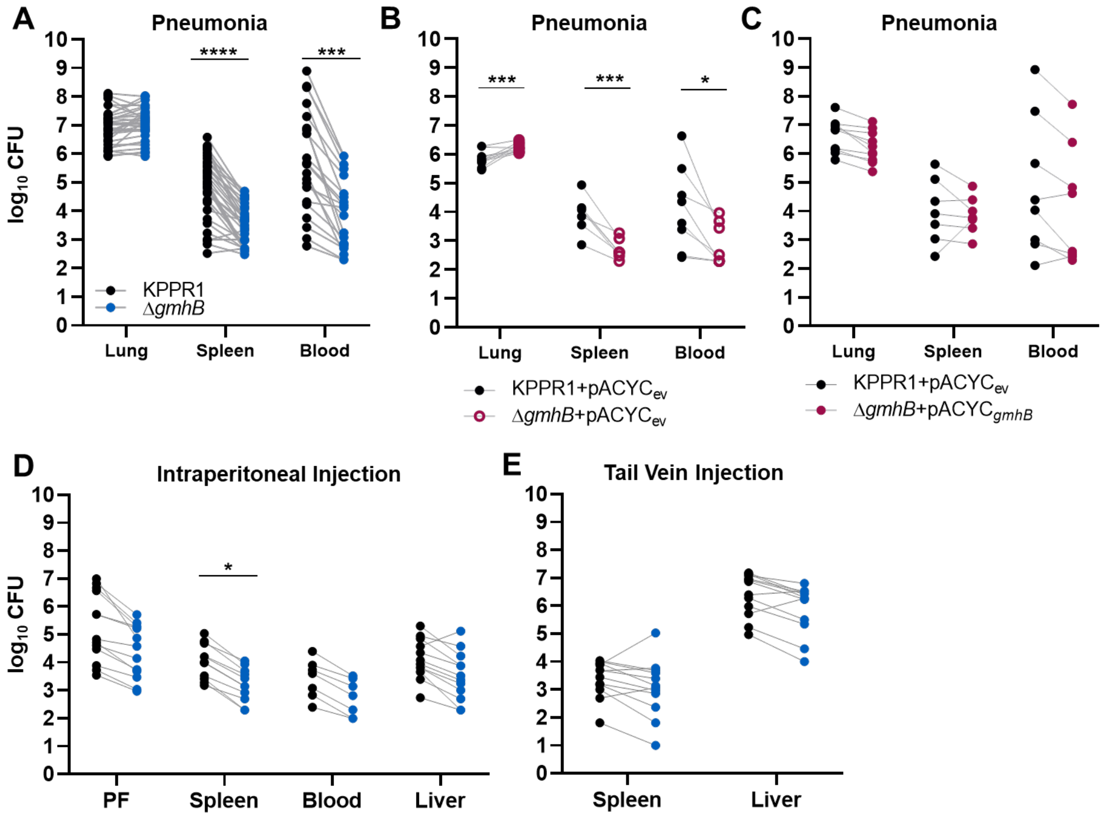

<u>Supplemental Figure 4.</u> <u>Bacterial burden summary for models of murine bacteremia.</u> In a model of bacteremic pneumonia, mice were retropharyngeally inoculated with $1x10^6$ CFU *K. pnuemoniae* (A-C). To initiate dissemination from a lung-independent site, $1x10^3$ CFU was administered to the intraperitoneal cavity (D). For modeling direct bacteremia requiring no dissemination, $1x10^5$ CFU was administered via tail vein injection (E). The 1:1 inoculum consisted of KPPR1:Δ*gmhB* (A, D, E), KPPR1:Δ*gmhB* carrying empty pACYC vector (ev; B), or $KPPR1_{ev}$:Δ*gmhB* with *gmhB* complementation provided on pACYC under control of the native *gmhB* promoter (Δ*gmhB*+$pACYC_{gmhB}$; C). Log_{10} CFU burden for each site at 24 hours post infection is displayed, corresponding to competitive indices in Figure 1. *$p<0.05$, **$p<0.01$, ***$p<0.001$, ****$p<0.0001$ by unpaired t test. For each group, $n \geq 7$ mice in at least two independent infections.

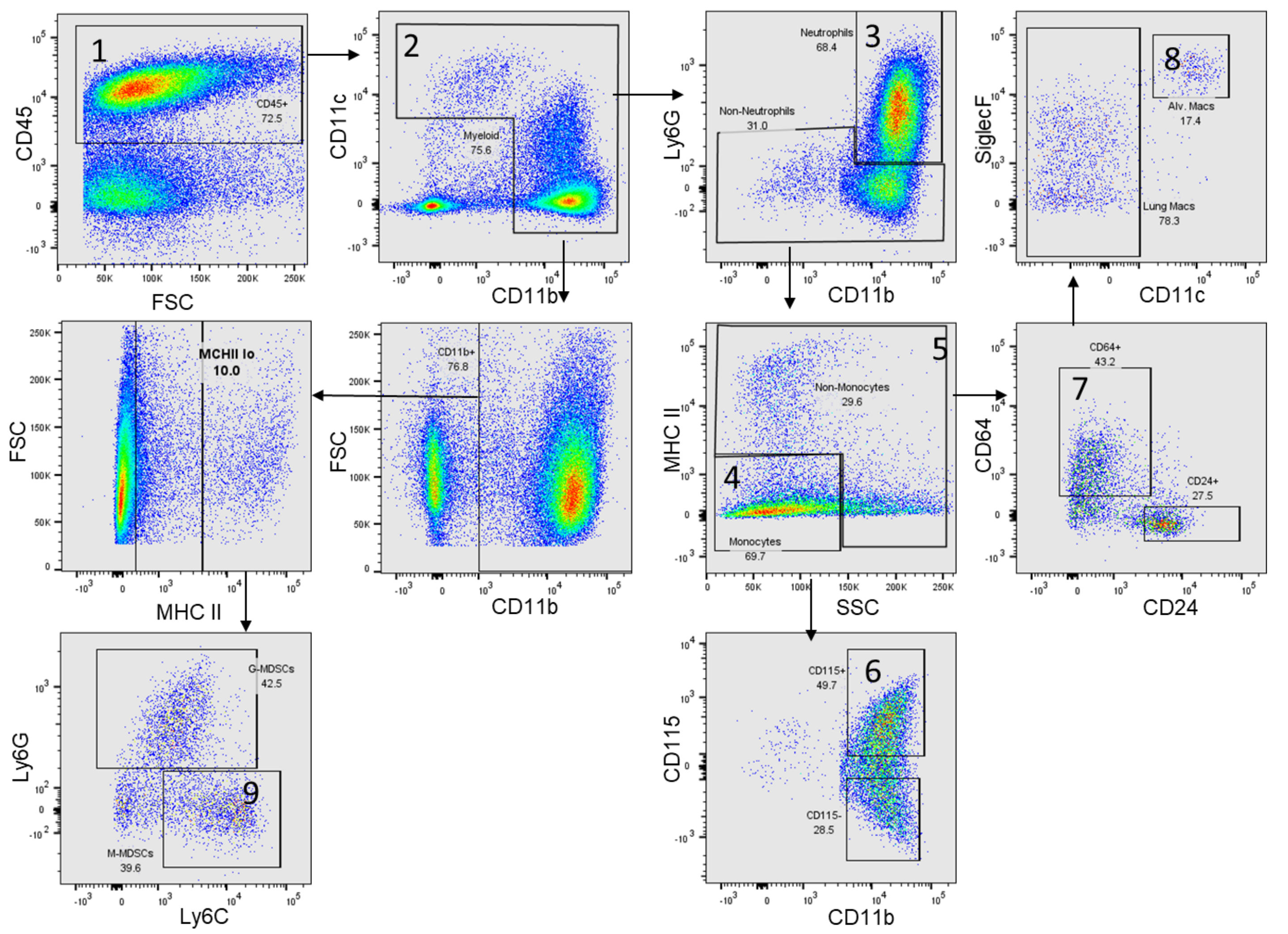

<u>Supplemental Figure 5.</u> <u>Gating Scheme for flow cytometry experiments.</u> Single cell suspensions were generated from collagenase digested lungs as described. Following this, cell viability was assessed via trypan blue exclusion and was >90% for all samples. Cells were subsequently gated as follows: $CD45^+$ (Gate 1), myeloid lineage cells: $CD11b/c^+$ (Gate 2), neutrophils: $Ly6G^+$ (Gate 3), putative monocytes: $MHCII^-$, SSC^{lo} (Gate 4) or macrophage and DCs: $MHCII^{+/-}$ SSC^{hi} (Gate 5), $CD115^+$ Monocytes (Gate 6), macrophages: $CD64^+$, $CD24^-$ (Gate 7), alveolar macrophages: $SiglecF^+$, $CD11c^+$ (Gate 8). M-MDSCs: $CD11b^+$, MCH^{lo}, $Ly6G^-$, $Ly6C^+$ (Gate 9).

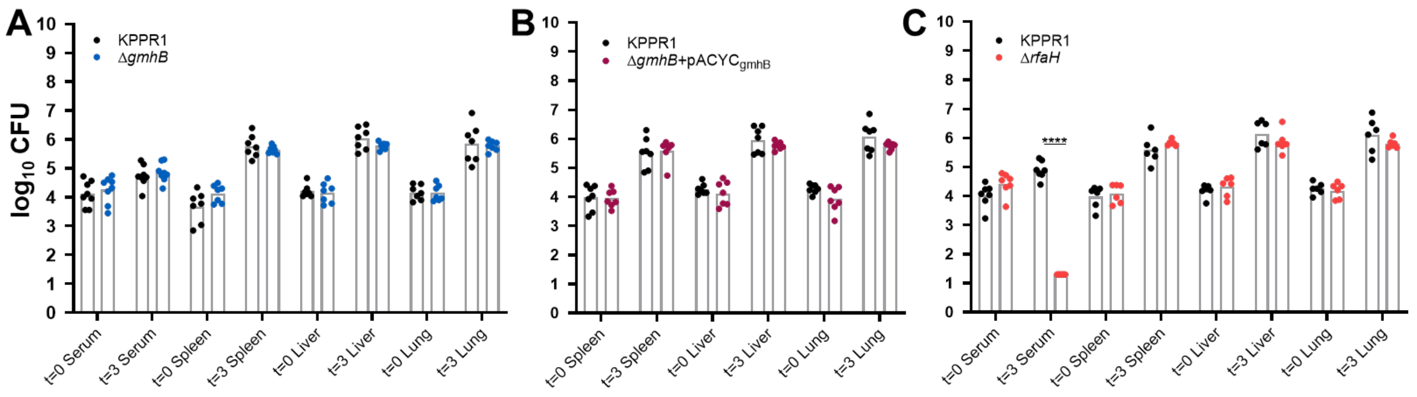

<u>**Supplemental Figure 6.** Bacterial counts from *ex vivo* killing assays.</u> *Ex vivo* competition assays were performed in human serum and uninfected murine spleen, liver, or lung homogenate using 1:1 mixture of KPPR1 and either Δ*gmhB* (A), Δ*gmhB*+pACYC$_{gmhB}$ (B), or Δ*rfaH* (C). Log$_{10}$ recovered CFU following 0 hours (t=0) and three hours (t=3) of incubation in specified condition is displayed, corresponding to competitive indices in Figures 3 and 4. ****$p<0.0001$ by unpaired t test, for each group, $n \geq 7$.

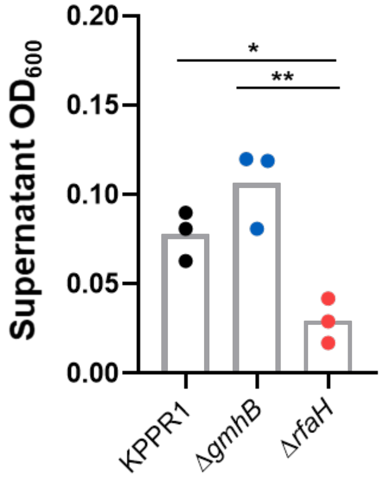

<u>Supplemental Figure 7.</u> <u>GmhB does not alter hypermucoviscosity.</u> To assess hypermucoviscosity, overnight cultures were pelleted at 5,000xg for 15 minutes and adjusted to an OD_{600}=1 in 1mL PBS. Normalized PBS suspensions were subsequently centrifuged at 1,000xg for 5 minutes and the OD_{600} of the upper 900µL of supernatant was measured from three biological replicates.

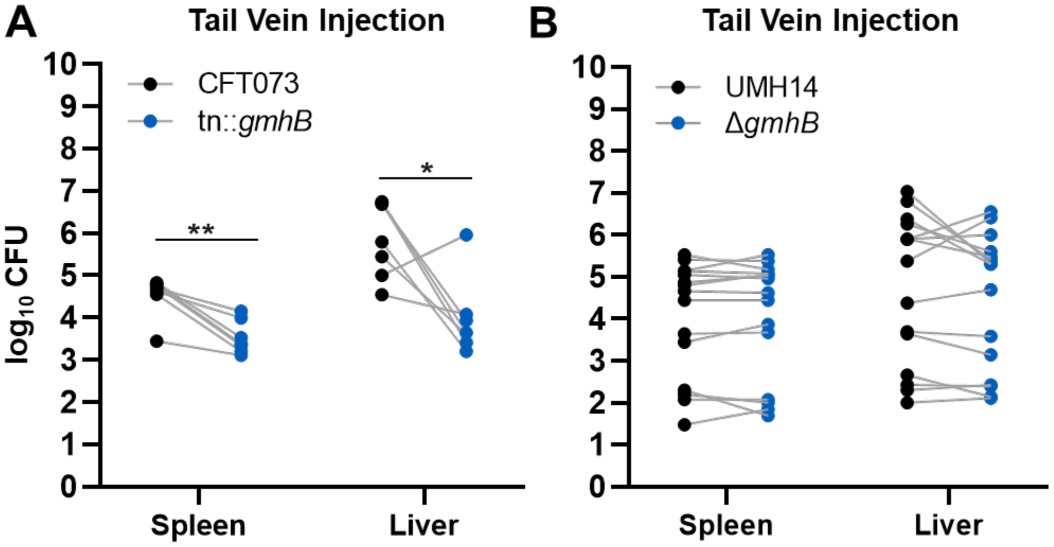

<u>**Supplemental Figure 8.** Bacterial burden summary for direct bacteremia with *E. coli* and *C. freundii.*</u>

In a model of direct bacteremia, $1x10^7$ CFU of *E. coli* CFT073 mixed 1:1 with CFT073:tn::*gmhB* (A) or *C. freundii* UMH14 mixed 1:2 with UMH14:Δ*gmhB* (B) was administered via tail vein injection. Log_{10} CFU burden for each site at 24 hours post infection is displayed, corresponding to competitive indices in Figure 6. *$p<0.05$, **$p<0.01$ by unpaired t test. For each group, $n\geq7$ mice in at least two independent infections.

SUPPLEMENTAL TABLES

Supplemental Table 1. Factors identified by transposon insertion site sequencing (InSeq) for involvement in late phases of *K. pneumoniae* bacteremia.

Locus ID (VK055_#)	Gene Name	Input:*Lcn2*$^{+/+}$ Lung		*Lcn2*$^{+/+}$:*Lcn2*$^{-/-}$ Lung		*Lcn2*$^{-/-}$ Lung:Spleen		GenBank Definition
		$\log_{10}$ ratio	q-value	$\log_{10}$ ratio	q-value	$\log_{10}$ ratio	q-value	
3924		0.978	1.000	0.876	0.221	25.500	1.84E-73	putative glycosylase
3792		1.138	0.160	0.872	0.137	25.467	1.45E-91	bacterial transferase hexapeptide family protein
4727		1.175	0.103	0.882	0.257	20.333	5.02E-70	ethanolamine ammonia-lyase, putative regulatory subunit
2040		1.054	1.000	1.083	0.883	20.000	7.62E-28	branched-chain amino acid transport system/permease component family protein
4483		1.011	1.000	1.022	1.000	16.909	3.66E-41	putative adhesin
2877	ulaA	0.979	1.000	1.043	0.858	16.556	1.52E-97	PTS ascorbate-specific subunit
2352	yaeD, gmhB	0.629	0.191	0.714	0.275	16.333	3.09E-11	D,D-heptose 1,7-bisphosphate phosphatase
3607	prlC	1.096	0.680	1.019	1.000	14.000	1.52E-32	oligopeptidase A
1436		0.752	0.053	1.051	1.000	13.800	4.01E-29	bifunctional enzyme and transcriptional regulator PutA transcriptional repressor, Proline dehydrogenase/pyrroline-5-carboxylate dehydrogenase
1606		0.918	0.941	0.825	0.371	12.875	1.77E-21	alpha-L-glutamate ligase, RimK family protein

4287	ptsP	0.976	1.000	1.092	0.268	12.419	4.21E-107	phosphoenolpyruvate-protein phosphotransferase
785	gcvA	0.727	0.191	0.767	0.222	12.286	8.48E-18	gcvA transcriptional dual regulator
4167	ubiX	1.084	0.611	0.937	0.784	12.048	1.49E-50	3-octaprenyl-4-hydroxybenzoate decarboxylase together with UbiG; flavy prenyltransferase
2933		1.171	0.074	0.997	1.000	11.926	6.05E-64	amino acid permease family protein; efflux transporter
2659	hpaB	1.175	0.205	0.910	0.626	11.824	4.37E-40	4-hydroxyphenylacetate 3-monooxygenase, oxygenase component
2390	dgt	1.025	1.000	1.063	0.745	11.538	5.90E-59	deoxyguanosinetriphosphate triphosphohydrolase
1770	pnuC	0.933	0.806	0.907	0.573	10.750	2.02E-41	nicotinamide mononucleotide transporter PnuC family protein
1674	exuT	0.884	0.698	0.905	0.819	10.500	1.82E-20	exuT hexuronate MFS transporter

Supplemental Table 2. Strains and plasmids used in this study.

Species	Strain	Modification	Reference (PMID)
Klebsiella pneumoniae	KPPR1	-	25291761
Klebsiella pneumoniae	KPPR1 pKD46	KPPR1 carrying the pKD46 plasmid	
Klebsiella pneumoniae	KPPR1 - Δ*gmhB*	KPPR1 with kanamycin resistance gene from pKD4 replacing the *gmhB* open reading frame	This study
Klebsiella pneumoniae	KPPR1 - Δ*gmhB*+$pACYC_{gmhB}$	ΔgmhB with *in trans* complementation of GmhB via pACYCgmhB	This study
Klebsiella pneumoniae	$KPPR1_{ev}$	KPPR1 carrying empty pACYC184 plasmid	This study
Klebsiella pneumoniae	KPPR1 - Δ$gmhB_{ev}$	ΔgmhB carrying empty pACYC184 plasmid	This study
Klebsiella pneumoniae	KPPR1 - Δ*rfaH*	KPPR1 with kanamycin resistance gene from pKD4 replacing the *rfaH* open reading frame	26060277
Klebsiella pneumoniae	KPPR1 - Δ*galU*	KPPR1 with kanamycin resistance gene from pKD4 replacing the *galU* open reading frame	33720976
Klebsiella pneumoniae	KPPR1 - Δ*4727*	KPPR1 with kanamycin resistance gene from pKD4 replacing the *4727* open reading frame	This study
Klebsiella pneumoniae	KPPR1 - Δ*2040*	KPPR1 with kanamycin resistance gene from pKD4 replacing the *2040* open reading frame	This study
Klebsiella pneumoniae	KPPR1 - Δ*4483*	KPPR1 with kanamycin resistance gene from pKD4 replacing the *4483* open reading frame	This study

Klebsiella pneumoniae	KPPR1 - Δ*ulaA*	KPPR1 with kanamycin resistance gene from pKD4 replacing the *ulaA* open reading frame	This study
Klebsiella pneumoniae	KPPR1 - Δ*prlC*	KPPR1 with kanamycin resistance gene from pKD4 replacing the *prlC* open reading frame	This study
Escherichia coli	CFT073	-	2182540
Escherichia coli	CFT073:tn::*gmhB*	CFT073 with a Mariner transposon insertion within the open reading frame of *gmhB*	This study
Citrobacter freundii	UMH14	-	30087402
Citrobacter freundii	UMH14:Δ*gmhB*	UMH14 with kanamycin resistance gene from pKD4 replacing the *gmhB* open reading frame	This study
	Plasmids		
	pKD46	Red recombinase expression plasmid of phage λ	10829079
	pKD4	Template plasmid carrying kanamycin resistance cassette	10829079
	pACYC184	Cloning vector conferring chloromphenicol resistance harboring restriction enzyme cut sites	
	pACYCgmhB	pACYC184 digested with BamHI and HindIII with 1,860bp KPPR1 region harboring gmhB open reading frames and predcited native promoter ligated into cut site by Gibson cloning	This study

Supplemental Table 3. Primers used in this study.

Primer Name	Primer Sequence (5'-3')
2040_flank_F	CTGACGAATGGACAACG
2040_flank_R	AAATCCTTGGCATGAGC
2040_internal_F	TAATATCCTGCACATAGGC
2040_internal_R	TTGGGATGATGGTTTCG
2040_pKD4_P1	TTAGTGTTTTTTCCGGTTGCGACGCATATCGATGGTTACTGCCGCAACGATAATGATGCCTTTAAGTGTAGGCTGGAGCTGCTTC
2040_pKD4_P2	ATGAGTAATATGAAATTAACGGCGGCTCCGGCCAGCGAGGGTTCCTCCTTCTTCGCTAATTTGCGATGGGAATTAGCCATGGTCC
4483_flank_F	GTTGGCGCACATTGATGG
4483_flank_R	AGCACTTTCGACAGAGATATGG
4483_internal_F	CGGTGTTGGGTGATATAGCG
4483_internal_R	ATAGCCGAAAGCGTCATGG
4483_pKD4_P1	GTGGTCGCGCTGGCCCTCGGCCTGATGGCGCAGGGGGCCATGGCGAAAACGCTGAATGTGGTAAGGTGTAGGCTGGAGCTGCTTC
4483_pKD4_P2	CCTGTTACTTCATGCTGTTAGCGATAAGCTCAACGTTGTGACGCATCATTTTGACGTAACTATCGATGGGAATTAGCCATGGTCC
4727_flank_F	GAAATCACTCGCATTTCCTTCC
4727_flank_R	TGATCCATGATGTGTCATCCC
4727_internal_F	AGGATGACGTGAACGAAACC

4727_internal_R	CAGATGCTCTGGAAGATAAGCC
4727_pKD4_P1	ATGAAACTAAAGACCACATTGTTCGGCAATGTTTATCAGTTTAAGGATGTAAAAGAGGTGCTGG CGTGTAGGCTGGAGCTGCTTC
4727_pKD4_P2	CCCCGCGTCATCAGAAGAACAGTGACGGATCGCCCGCCCGTTTGGTCAGGCGACCATTTGCC ATAATGGGAATTAGCCATGGTCC
gmhB_flank_F	TATAGGGAGCTCTCCGGGAA
gmhB_flank_R	GCGGAAACATGCGATACTAGC
gmhB_internal_F	CTCTCCGGGAATGACAAGC
gmhB_internal_R	TGAAACGCTGACCGAATGG
gmhB_pKD4_P1	TTATTTTTGCTGCTTTTTAATCGCCGCCGGCAGCTCTGCCAGGCTATTTAGCACCCAATCCGCC GGTGTAGGCTGGAGCTGCTTC
gmhB_pKD4_P2	AGCAAACCGGTGGCAAAGTCAGTACCCGCAATTTTTCTCGACCGTGACGGCACTATTAATGTC GAATGGGAATTAGCCATGGTCC
prlC_flank_F	GCCCGTCATTGAAATTCCC
prlC_flank_R	TTCATCAATTAAGCAGATCTTCACG
prlC_internal_F	GCTTCTATCTCGACCTGTATGC
prlC_internal_R	CTGCTCCGGTTTGTACTCC
prlC_pKD4_P1	ATGACCAATCCATTATTGACGCCTTTCTCCCTGCCGCCTTTTTCTGCGATCAAACCAGAGCACG TGTGTAGGCTGGAGCTGCTTC
prlC_pKD4_P2	TCAGCCTTTGATCCCGTAATGCTCCAGCATCGCGTCCAGCTGCGGCTCGCGCCCGCGGAAGC GTTATGGGAATTAGCCATGGTCC
ulaA_flank_F	GTTACCACATACAGCCAGAATACG

ulaA_flank_R	CGAGAAATGCTGAGCGTAAGG
ulaA_internal_F	TTCAACCATGCCCATCACC
ulaA_internal_R	CAGGCTGGCTCAATATCTTCC
ulaA_pKD4_P1	GCCCGTTACCACATACAGCCAGAATACGTACGGTCATAATCAGAACTCCTTATTGGGCAGAGG CCGTGTAGGCTGGAGCTGCTTC
ulaA_pKD4_P2	TATGGCAATCCTCTACAACATCTTTACCGTTTTCTTTAATCAGGTGATGACCAATGCCCCGCTG CATGGGAATTAGCCATGGTCC
gmhB_pACYC_seq_F	GCCGAAACAAGCGCTCATGAG
gmhB_pACYC_seq_R	TCAGAGCAAGAGATTACGCGC
gmhB_pKD4_UMH14_P1	GTGGCAAAGTCTGTACCCGCAATTTTTCTCGACCGTGACGGTGTAGGCTGGAGCTGCTTC
gmhB_pKD4_UMH14_ P2	TGCATTTTTGTTTCAACCGCTCACCTTTTACCCATAACGCATATGAATATCCTCCTTAGT
gmhB_UMH14_seq_F	CTGTCAGGTACGGTCGAAGCC
gmhB_UMH14_seq_R	CGGCTGTTTGTGTGAAGTGATTC

Supplemental Dataset 1. Selection Strategy for candidate dissemination factors.

Contains two tabs: “Supplementary Data” contains number of insertions, insertion counts, ratio and q-value for each gene and each comparison used for selection. “Selection” describes the criteria for considering a gene to be significantly associated with lung dissemination.
